# The Role of the Thioredoxin Detoxification System in Cancer Progression and Resistance

**DOI:** 10.3389/fmolb.2022.883297

**Published:** 2022-05-19

**Authors:** Mirna Jovanović, Ana Podolski-Renić, Mikhail Krasavin, Milica Pešić

**Affiliations:** ^1^ Department of Neurobiology, Institute for Biological Research “Siniša Stanković”- National Institute of Republic of Serbia, University of Belgrade, Belgrade, Serbia; ^2^ Organic Chemistry Division, Institute of Chemistry, Saint Petersburg State University, Saint Petersburg, Russia

**Keywords:** Trx, TrxR, RONS, oxidative stress, antioxidative defence, resistance to therapy

## Abstract

The intracellular redox homeostasis is a dynamic balancing system between the levels of free radical species and antioxidant enzymes and small molecules at the core of cellular defense mechanisms. The thioredoxin (Trx) system is an important detoxification system regulating the redox milieu. This system is one of the key regulators of cells’ proliferative potential as well, through the reduction of key proteins. Increased oxidative stress characterizes highly proliferative, metabolically hyperactive cancer cells, which are forced to mobilize antioxidant enzymes to balance the increase in free radical concentration and prevent irreversible damage and cell death. Components of the Trx system are involved in high-rate proliferation and activation of pro-survival mechanisms in cancer cells, particularly those facing increased oxidative stress. This review addresses the importance of the targetable redox-regulating Trx system in tumor progression, as well as in detoxification and protection of cancer cells from oxidative stress and drug-induced cytotoxicity. It also discusses the cancer cells’ counteracting mechanisms to the Trx system inhibition and presents several inhibitors of the Trx system as prospective candidates for cytostatics’ adjuvants. This manuscript further emphasizes the importance of developing novel multitarget therapies encompassing the Trx system inhibition to overcome cancer treatment limitations.

## Introduction

Cancer is a complex and heterogeneous disease, with more than 18 million diagnosed cases per year, and more than nine million cancer-related deaths worldwide. With the ongoing rate of progression, the annual number of cases will rise to 29.5 million by the year 2040, while 16.5 million people will die of cancer (NIH National Cancer Institute, 2020). Despite tremendous efforts and resources invested in cancer research in the past, with significant progress made in unraveling the biology of cause and progression, the constant development of new modalities of diagnosis and therapies, cancer appears unconquerable in the vast majority of cases. The reason why treating cancer is highly challenging lays behind the fact that cancer is developed by fast-evolving, highly adaptable to change, and highly heterogenic malignant cells. The canonical approach to clinical cancer treatment consists of surgical resection, chemotherapy, and radiotherapy when possible. Resistance to drugs remains a major obstacle to effective chemotherapy treatment. Common mechanisms of drug resistance are the overexpression of efflux pumps (Cojoc et al., 2015; Fletcher et al., 2016), change in the expression of the target molecule (Benhar et al., 2016; Yan et al., 2019), boosted DNA repair machinery (Bao et al., 2006; Cojoc et al., 2015; Lee, 2016), apoptosis evasion (Hanahan and Weinberg, 2011) and increase in resilience to reactive oxygen and nitrogen species (RONS) (Landriscina et al., 2009; Traverso et al., 2013; Marengo et al., 2016). A strategy in developing new chemotherapeutical approaches is adding adjuvant compounds to cytostatic in clinical practice, thus tackling several pro-survival mechanisms at the same time. Given the dynamic nature of cancer, the multitarget approach gives hope in arresting tumor growth and progression.

It is a well-known fact that antioxidant detoxifying systems are of fundamental significance for cancer cell survival upon exposure to an anti-cancer drug. Therefore, the simultaneous shutdown of these defense systems can provide therapy success, lower systemic toxicity, and influence the patient’s life quality (Benhar et al., 2016; Yan et al., 2019). The thioredoxin (Trx) system is one of the vital threads in the cell’s antioxidant detoxification network, responsible for the preservation of redox homeostasis. The over-activated Trx system, as a widespread event in cancer cells (Table 1), is correlated with poor clinical prognosis and poor response to chemo- and radiotherapy. In this review, we present some of the highlights in research emphasizing the Trx system’s role in cancer progression and resistance to therapy, as well as exploration of the system as a target in developing novel therapeutics.

**TABLE 1:** The role of the Trx system in specific cancer types.

Breast	cancer progression, drug ressistance, migration, and invasion	Iwao-Koizumi et al. (2005), Kim et al. (2005), Harris et al. (2015), Bhatia et al. (2016), Rodman et al. (2016), Raninga et al. (2020)
Cervical	drug resistance	Du et al. (2012), Liu et al. (2019), Zhu et al. (2019b)
Colon and colorectal	cancer progression, drug resistance	Berggren et al. (1996), Raffel et al. (2003), Du et al. (2018), Han et al. (2019), Marmol et al. (2019)
Gastric and gastrointestinal	cancer progression, drug resistance	Pessetto et al. (2013), He et al. (2019), Zhang et al. (2019), Wu et al. (2020)
Glioma	cancer progression, drug resistance, migration, and invasion	Haapasalo et al. (2003), Kemerdere et al. (2013), Zhang et al. (2017b), Erdi et al. (2018), Haas et al. (2018), Jovanovic et al. (2020a), Yao et al. (2020)
Hepatocellular	cancer development and progression	Zheng et al. (2018), Lee et al. (2019), McLoughlin et al. (2019)
Head and neck	cancer progression, drug resistance	Hoshikawa et al. (2010), Hoshikawa et al. (2011), Zhu et al. (2011), Sobhakumari et al. (2012), Indo et al. (2014), Roh et al. (2017a)
Lung	cancer development and progression, drug resistance	Kakolyris et al. (2001), Soini et al. (2001), Bjorkhem-Bergman et al. (2002), Yoo et al. (2006), Dai et al. (2013), Fan et al. (2014), Li et al. (2016), Hou et al. (2018), Zhu et al. (2019a)
Leukemia	cancer development, drug resistance	Sasada et al. (1996), Olm et al. (2009), Fidyt et al. (2019), Xu et al. (2019), Cirri et al. (2020), Noura et al. (2020)
Medulloblastoma	cancer progression	Yao et al. (2020)
Melanoma	cancer development	Sachweh et al. (2015), Obrador et al. (2019)
Myeloma	drug resistance	Raninga et al. (2015), Raninga et al. (2016)
Mesothelioma	drug resistance	You and Park, (2016)
Neuroblastoma	migration and invasion	Farina et al. (2001)
Ovarian	drug resistance	Wang et al. (2015), Landini et al. (2017)
Pancreatic	drug resistance	Arnold et al. (2004), Cai et al. (2020)
Thyroid	cancer progression	Morrison et al. (2014)

## Oxidative Stress and Antioxidant Defense Systems

**FIGURE 1:**
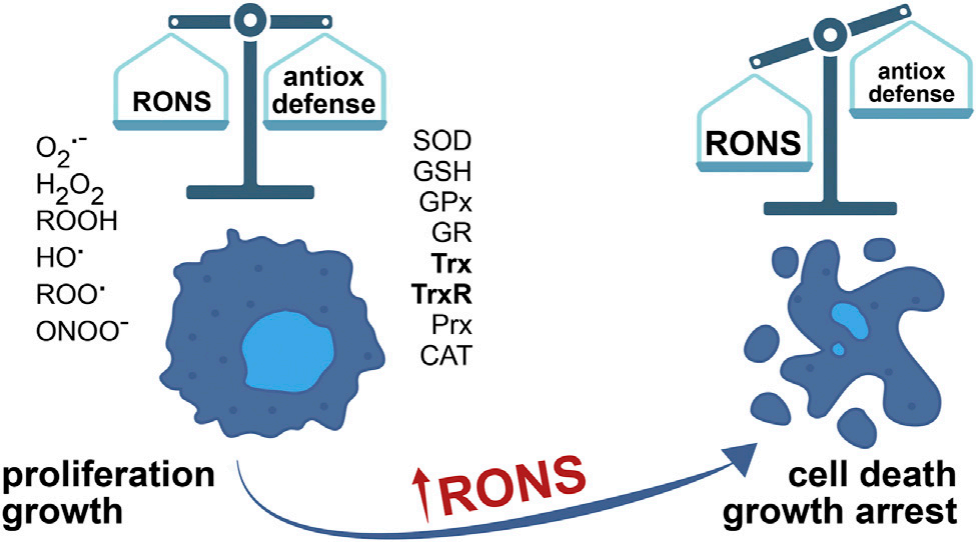
The balance between RONS and the antioxidant defense system determines cell faith**.** The concentration of RONS is elevated in cancer cells, due to aberrant metabolism adjusted to accelerated growth and proliferation. To survive, cancer cells over-express enzymes of antioxidant defense. What’s more, RONS in cancer cells have an important part in tumor growth and promotion, in all stages of tumor development. However, further increase in RONS, by inhibition of the antioxidant detoxification systems, for example, causes cell death and inhibits tumor growth.

Intracellular reactive oxygen species (ROS) are intermediate products of oxidative metabolism and ROS-generating enzymes; examples of intracellular ROS are superoxide ion (O_2_^−^), hydrogen peroxide (H_2_O_2_), organic hydroperoxides (ROOH), hydroxyl radical (HO^−^), peroxyl radical (ROO^·^), and others (Figure 1). Reactive nitrogen species (RNS) are a group of diverse compounds, with one unifying property of nitrogen monoxide (NO) derivatives. As a signaling molecule, NO participates in S-nitrosylation, a fast and reversible change in protein structure and activity. Superoxide ion easily reacts with NO, generating peroxynitrite (ONOO^−^) as a product ONOO^−^ causes oxidation, nitrosation (addition of NO), or nitration (addition of NOO) to biomacromolecules. Nitric oxide can also be a source of O_2_^−^. ROS and RNS are considered an intricately connected and dynamic group of highly reactive chemical species, described under a single acronym–RONS.

Oxidative stress is a condition of misbalance between the rate of RONS production and the activities of antioxidant defense systems, where the production of RONS exceeds the capacity of cells to neutralize these harmful chemical species (Figure 1). Besides the damage they cause to the cell, RONS have a physiological role as well, as part of redox homeostasis maintenance systems. In healthy functional cells, in low concentration and controlled synthesis, RONS act as signaling molecules stimulating proliferation and cell survival (Trachootham et al., 2009; Sies and Jones, 2020; Tretter et al., 2021). Antioxidant systems, comprised of enzymes and small molecules, control and regulate RONS concentration inside the cell (Figure 1). Moderately elevated levels of RONS cause cell impairment by lipid peroxidation of membrane systems, oxidation of proteins, and nucleic acids (Ryter et al., 2007). DNA damage increases the mutagenesis rate and increases the probability of cell malignant transformation. High concentrations of RONS induce irreversible cellular malfunction, ultimately causing senescence and/or cell death (Ryter et al., 2007; Trachootham et al., 2009). Crucial signaling pathways responsive to change in redox state, capable of triggering signals for cell death initiation, are ASK1/JNK and ASK1/p38 MAP signaling cascades (Mieyal et al., 2008).

**FIGURE 2:**
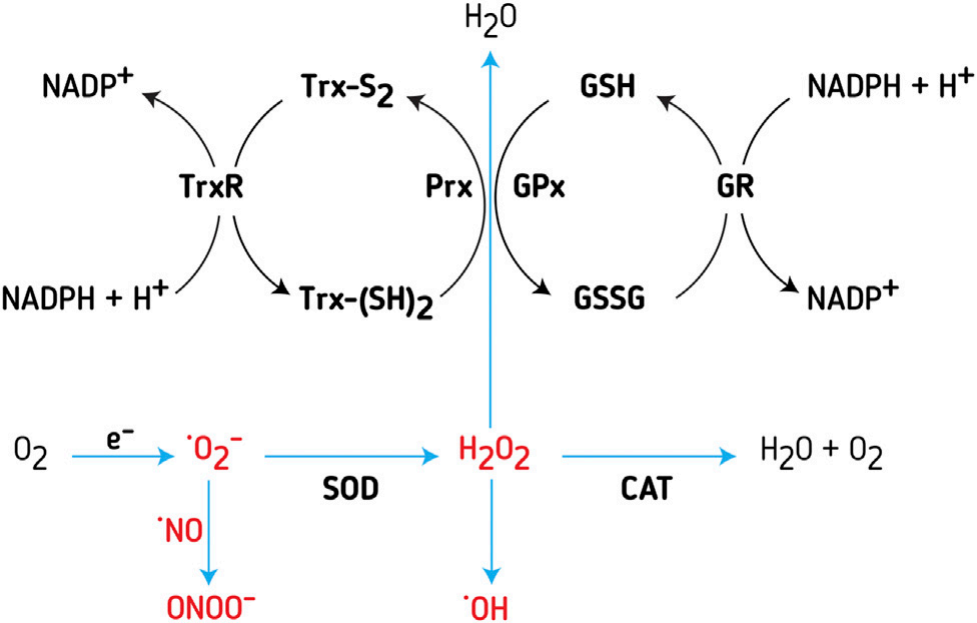
Redox systems ‐ Trx and GSH systems, are important regulators of intracellular RONS concentration**.** Major sources of RONS inside the cell are the electron transport chain (ETC) in mitochondria and NADPH oxidases (NOX). Superoxide anion (O_2_^−^) is the main free-radical deriving from ETC; due to lack of stability, the molecule is relatively easily transmuted to hydrogen peroxide (H_2_O_2_) or peroxynitrite (ONOO^−^). Superoxide dismutase (SOD) is an enzyme catalyzing the transmutation of 2O^−^ to H_2_O_2_. Further on, H_2_O_2_ is neutralized by several different enzymes and enzyme systems. The most potent is peroxiredoxins (Prx), a cysteine-dependent peroxidase that reacts with H_2_O_2_. Thioredoxin (Trx) reduces Prx, while thioredoxin reductase (TrxR) reduces oxidized Trx, with NADPH as an electron donor. Glutathione peroxidase (GPx) uses reduced glutathione (GSH) for neutralizing hydrogen peroxide. In turn, glutathione reductase (GR) reduces oxidized glutathione (GSSG), with NADPH as an electron donor. Another enzyme, involved in the regulation of H_2_O_2_ concentration, found mainly in peroxisomes and cytosol, is catalase (CAT).

Uncontrolled release of RONS causes irreversible damage, and therefore cells tend to limit or neutralize RONS in strive to preserve the functional integrity (Figure 1). Cellular detoxifying systems encompass antioxidant enzymes and redox molecules: superoxide dismutase (SOD), glutathione (GSH), glutathione peroxidases (GPx), glutathione reductase (GR), Trx, and thioredoxin reductase (TrxR), peroxiredoxins (Prx) and catalase (CAT) (Figure 2). By catalytic activity of SOD, O_2_^−^ transforms into H_2_O_2_ (Figure 2). SODs accelerate spontaneous dismutation of O_2_^−^ by the factor of 10^3^. Humans have three types of SOD: cytoplasmic SOD1 and extracellular SOD3, with CuZn cofactor, and mitochondrial SOD2, with Mn cofactor. H_2_O_2_ is less reactive than O_2_^−^, less toxic and it can diffuse through membranes (Gadjev et al., 2008), which makes it a suitable signaling molecule–it reacts with Cys residues of redox-sensitive proteins, particularly Prx allowing the formation of disulfide bridges between two Cys or between Cys and GSH, in the process of protein S-glutathionylation (Cai and Yan, 2013). The Prx-based redox mechanism seems to be responsible for the majority of thiol oxidations (Stocker et al., 2018). Namely, the catalytic Cys of Prx reacts with H_2_O_2,_ and then instead of reacting with a second Cys in Prx, the intermediate-sulfenic acid interacts with a Cys residue of a target protein thus forming a disulfide bridge. A second Cys of the target protein attacks this disulfide, release the reduced Prx and forms a disulfide bond in the target protein (Rhee et al., 2018). Prxs are the most sensitive signaling transducers of H_2_O_2_ mediated redox signaling although Prx-independent H_2_O_2_ signaling mechanisms can also exist (Ulrich and Jakob, 2019).

H_2_O_2_ through the Fenton reaction may become a source of HO^−^ (Imlay et al., 1988), another highly unstable and toxic species (Figure 2). This is why cells tend to sequester the Fenton metals to reduce the HO^−^. In the cell, Prx, GPx, and CAT are involved in H_2_O_2_ intracellular homeostasis (Figure 2). ONOO^−^ is removed by GSH and other thiols, as well as Mn and Fe porphyrins. GSH also removes various reactive species, being oxidized into GSSG. GR reduces GSSG back to GSH, recovering the pool of available antioxidants (Figure 2). In a reduced state, Trx reduces Prx, while TrxR reduces the oxidized Trx (Figure 2).

The nuclear factor erythroid 2-related factor 2 (Nrf2) transcription factor regulates the expression of most antioxidant enzymes. In homeostatic redox conditions, Nrf2 has a half-life of approximately 20 min and is continually degraded by ubiquitination, with Kelch-like ECH-associated protein 1 (Keap1) as its marker for degradation (Itoh et al., 2010). In oxidative stress, RONS oxidize Keap1 Cys residues, the protein dissociates from Nrf2 and Nrf2 degradation ceases; Nrf2 is free to translocate into the nucleus, where it forms dimers with Maf proteins and interacts with antioxidant response element (ARE) sequence (Itoh et al., 2010). Nrf2 protects cells from oxidative damage through the expression of target genes involved in RONS detoxification, such as antioxidant enzymes *SOD*, *CAT*, *PRX*, *GR*, *TRX,* and *TRXR*, as well as genes involved in drug metabolism/transport - *ABCC1* and *ABCG2* (Jaramillo and Zhang, 2013; Kim et al., 2019). A large number of studies have shown that Nrf2 is related to cancer initiation (Jaramillo and Zhang, 2013; Rojo de la Vega et al., 2018).

## Role of Oxidative Stress in Tumor Development

RONS activate pro-tumorigenic signalization, increase survival and proliferation, and cause DNA damage and genetic instability (Moloney and Cotter, 2018; Hayes et al., 2020). Constitutively elevated RONS, due to the increased rate of metabolism, support fast proliferation and growth and contribute to both tumorigenesis and tumor progression (Hayes et al., 2020). To evade senescence, apoptosis, necrosis, and ferroptosis, cancer cells adapt to ubiquitously elevated RONS levels. Several processes lead to this adaptation: (I) increased expression of detoxifying antioxidant enzymes, such as Trx (Janssen et al., 1999; Janssen et al., 2000; Arner and Holmgren, 2006; McLoughlin et al., 2019), (II) inactivation of enzymes for H_2_O_2_ removal (Toledano et al., 2010), (III) inactivation of tumor suppressors (Leslie et al., 2003), and (IV) increased synthesis of small antioxidant molecules, such as GSH (Traverso et al., 2013). Oxidative stress is one of the main culprits for cancer cells being highly inefficient in metastasis (Piskounova et al., 2015; Gill et al., 2016). When entering the bloodstream, malignant cells are exposed to increased oxygen levels: oxygen level is 2–5% in physoxic tissue (in cancer tissue it can be lower than 1%), while in arterial bloodstream O_2_ is around 10% (McKeown, 2014). In such an environment, most of the cells with metastatic potential die due to oxidative stress. Cells successful in inhabiting distant tissues and organs have highly active transcription factors regulating the expression of antioxidant defense system proteins, as RONS neutralization is of pivotal significance for metastatic cell survival (Gill et al., 2016). Therefore, it is not surprising that the antioxidant treatment caused tumor progression and increased the number of metastatic cells in lung cancer and melanoma (Sayin et al., 2014; Piskounova et al., 2015; Obrador et al., 2019). In contrast, other authors reported that antioxidant treatment and RONS reduction are capable of inhibiting metastasis and decreasing metastatic cancer cell potential (Sotgia et al., 2011; Buric et al., 2019). It is well known that oxidative stress is a “double-edged sword” in cancer biology and treatment (Acharya et al., 2010; Farhood et al., 2019).

## Significance of Trx System in Redox Balance Maintenance

Thiol groups (SH-), such as those present in Cys residues of proteins or peptides, are of high importance for metabolic, signaling, and transcriptional processes in mammalian cells. Redox reactions of these chemical groups are fast and reversible, so proteins regulated by Cys residues are capable of quick response to changes in the intra- and extracellular environment and cell signaling. Thiol groups are frequent targets of RONS oxidation, which is why systems responsible for redox balance maintenance are of imponderable relevance for RONS detoxification in oxidative stress. The fundamental role of thiol buffer systems, comprised mainly of GSH and Trx systems, is the maintenance of redox balance in the cell. The two systems overlap in function and compensate for each other in case one of the two fails to provide antioxidant protection (Du et al., 2012). Decreased activity of one of the systems most often leads to the upregulation of the other (Benhar et al., 2016; Yan et al., 2019).

GSH is a small tripeptide molecule and the most abundant antioxidant in the cell. GSH is present in all cell types and all cell compartments, and by rough estimate, more than 10% of it is found in mitochondria, where it counteracts RONS from the electron transport chain. GSH is involved in sustaining redox homeostasis, detoxification of xenobiotics and it is a reservoir of intracellular Cys. GSH system, besides GSH, includes enzymes involved in the synthesis of GSH, such as γ-glutamyl-cysteine ligase and γ-glutamyl-transpeptidase, and enzymes relying on GSH to conduct their role. The family of glutathione-S-transferase (GST) enzymes detoxifies xenobiotics by conjugating them to GSH. GPx, as mentioned earlier, neutralizes H_2_O_2_ using GSH, while producing oxidized GSSG and water. Glutaredoxins (Grx) are a class of small redox enzymes, neutralizing RONS at the expense of GSH, participating in redox signalization, and regulating metabolic pathways (Lillig et al., 2008). GR reduces GSSG to GSH, with nicotinamide adenine dinucleotide phosphate (NADPH) as an electron donor. As a consequence of oxidative stress, cancer cells frequently have increased levels of GSH, as well as GSH synthesizing enzymes (Traverso et al., 2013). Cancer cells demonstrate higher expression of proteins of the GSH synthetic pathway, GR, and Grx (Enoksson et al., 2005; Zhao et al., 2009; Desideri et al., 2019). Increased GSH contributes to tumor progression, promotes cancer cell survival, and affects adaptation and resistance to therapy (Traverso et al., 2013; Harris et al., 2015).

**FIGURE 3:**
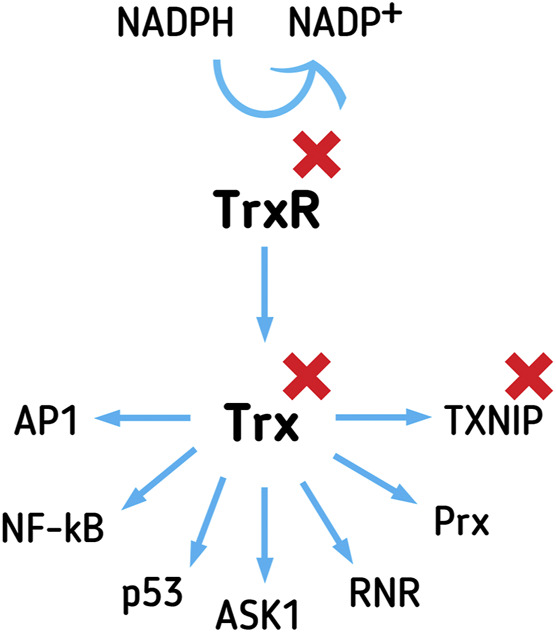
The Trx system affects gene expression, apoptosis, proliferation, and oxidative and xenobiotics defense, through interactions with versatile executing proteins. Trx system comprises Trx, TrxR, and NADPH. TrxR reduces Trx, using NADPH as an electron donor. TXNIP inhibits Trx function in the cell. Trx affects the DNA binding affinity of transcription factors, such as AP1, p53, and NF-κB. Trx inhibits apoptosis-promoting protein ASK-1 and promotes proliferation through RNR activation. Trx reduces Prx, enabling it to provide antioxidant detoxification of peroxides inside the cell. Inhibitors of the Trx system are attractive targets for the development of novel anticancer drugs.

Trx system comprises Trx, TrxR, thioredoxin-interacting protein (TXNIP), and NADPH (Figure 3). Protein isoforms of the Trx system, present in most human cells, are Trx1 and TrxR1 in the cytoplasm and nucleus, and Trx2 and TrxR2 in the mitochondria. Apart from intracellular Trx and TrxR, the extracellular activity of secreted Trx system proteins has also been detected (Soderberg et al., 2000; Pekkari et al., 2001; Matsuo and Yodoi, 2013), in the serum of healthy individuals. The secreted proteins play a beneficial role in overcoming inflammation, and the truncated form of Trx–Trx80 (truncated to around 80 amino acids) acts as a monocyte growth factor. Furthermore, the increased secretory release of Trx and TrxR into peripheral blood has been confirmed for tumor cells as well, with the proposed role of protecting tumor cells from extracellular oxidation and the immune system (Soderberg et al., 2000). Using NADPH as an electron donor for reduction reaction, TrxR reduces Trx (Zhong et al., 2000; Fritz-Wolf et al., 2011). Trx and TrxR activity is implicated in gene activation, cell cycle, and especially in cell protection and survival (Arner and Holmgren, 2000; Gromer et al., 2004; Karlenius and Tonissen, 2010). Aside from the expression regulation, TXNIP is another line of Trx activity control, acting as an inhibitor of Trx (Patwari et al., 2006). TXNIP is a tumor suppressor, commonly silenced in cancer cells, by genetic or epigenetic events (Morrison et al., 2014; Zhang P. et al., 2017; Noura et al., 2020).

Trx1 is a small, 12 kDa, multifunctional disulfide reductase, with redox-active, Cys containing, motifs (-CXXC-), existing in the oxidized (disulfide) or reduced (dithiol) state. Trx, reduced by TrxR1, in a dithiol form of the active site, transfers reducing equivalents to disulfides within the target molecules, reducing protein disulfides and acting as a hydrogen donor to proteins (Holmgren, 1989; Arner and Holmgren, 2000). In the nucleus, the protein binds directly to different transcription factors, such as p53, NF-κB, and activator protein (AP1) (Lillig and Holmgren, 2007), thus modulating the DNA-binding affinity of the interacting transcription factors (Figure 3). In the cytoplasm, Trx1 interacts with apoptosis signal-regulating kinase 1 (ASK-1, Figure 3), inhibiting its function as a promoter of the JNK/p38 apoptosis signaling pathway (Fujino et al., 2006). Trx is a hydrogen donor for ribonucleotide reductase (RNR, Figure 3) (Holmgren, 1989), a crucial enzyme of deoxyribonucleotides synthesis, and a key limiting step of proliferation. Prx, important in the detoxification of H_2_O_2_, lipid peroxides, and peroxynitrite, is another protein dependent on Trx reduction (Figure 3) (Hanschmann et al., 2013). There are reports of high expression of Prx in different types of primary tumors (Fourquet et al., 2008; Kim and Jang, 2019).

TrxR catalyzes the reduction of Trx disulfides, through the selenocysteine (Sec) C-terminal active site (Arner and Holmgren, 2000; Zhong et al., 2000; Fritz-Wolf et al., 2011). Mammalian TrxR1 is, compared to the enzyme in bacteria, plants, and fungi, a large enzyme. It is around 114 kDa in size and is an NADPH homodimeric selenoflavoenzyme. The two identical subunits of the enzyme, 57 kDa each, are oriented head-to-tail, where the N-terminus of one subunit is in close proximity to the C-terminus of the other subunit. Electrons from NADPH are transmitted to the flavin adenine dinucleotide (FAD) domain, further on, electrons travel to N-terminal redox pair C59/C64 of one subunit, from where they are transmitted to C-terminal Cys497/Sec498 redox pair of the other subunit (Zhang J. et al., 2017). Apart from Trx1, TrxR1 also reduces, disulfide protein isomerases, important for posttranscriptional bending and processing of proteins (Lothrop et al., 2009). Additionally, TrxR1 recycles antioxidant molecules such as dehydroascorbate, lipoic acid, and ubiquinone (Kalinina et al., 2008). Following the variable oxidative and hypoxic stress in the tumor environment, the expression of Trx system proteins increases (Karlenius and Tonissen, 2010). Redox state post-transcriptionally regulates the expression of TrxR1, allowing fast change in expression in response to alterations in the intracellular environment (Rundlof et al., 2001). The exceedingly active Trx system detoxifies RONS, thus protecting transformed cells from oxidative stress-induced cell death (Gromer et al., 2004). Increased expression and/or activity of Trx system proteins have been reported for breast, lung, thyroid, liver, prostate, pancreas, and colon cancer, melanoma, glioma, medulloblastoma, and others (Table 1). Overexpression of Trx correlates with aggressive tumors, poor prognosis, and a lower survival-rate in patients (Kakolyris et al., 2001; Raffel et al., 2003; Zhu et al., 2011). Both GSH and Trx actively affect metastasis and tumor progression (Harris et al., 2015). Trx1 increases the mobility and invasiveness of cancer cells (Bhatia et al., 2016). Trx promotes matrix metalloproteinase −2 and −9 (Farina et al., 2001), enzymes with a role in the degradation of the extracellular matrix, and the main culprits in acquiring and maintaining metastatic potential in cancer cells. Therefore, targeting the Trx system as a whole or its key components is considered a well-justified anticancer strategy (Table 1).

## Correlations of the Trx System With Cancer Drug Resistance

Drug resistance remains one of the major causes of cancer treatment failure, especially in the case of advanced and disseminated cancers (Stankovic et al., 2019). There are two types of drug resistance: inherent drug resistance usually observed when cancer arises from a tissue that is already physiologically highly protected from toxins or acquired drug resistance when initially sensitive cancer cells develop resistance to applied therapy. However, a phenomenon described as multidrug resistance (MDR) implies that cancer cells can become not only resistant to the applied anticancer drug but develop simultaneous resistance to a number of structurally and functionally unrelated chemotherapeutic agents (Assaraf et al., 2019). MDR can arise from a number of underlying mechanisms. One of the most extensively studied mechanisms of MDR is the overexpression of ATP-Binding Cassette (ABC) transporters. These transporters reduce intracellular drug concentration, leading to a decrease in their cytotoxicity (Dinic et al., 2018). It has been also shown that MDR can result from altered drug target, enhanced DNA damage repair, altered thiol or redox homeostasis, impaired induction of apoptosis, and altered metabolism of MDR cancer cells (Valente et al., 2021). Moreover, there is increasing evidence that the entire composition of the tumor microenvironment, including fibroblasts or immune cells, has a significant impact on chemotherapy success (Petanidis et al., 2019).

Mechanisms of cancer cell adaptation to oxidative stress and drug resistance are intersecting (Pennington et al., 2005; Landriscina et al., 2009). The high RONS production in cancer cells induces an antioxidant adaptive response, triggering a state of altered redox signalization and activation of pro-survival mechanisms. The signaling proteins govern intra- and intercellular communication and have pivotal significance in proliferation and determining cell faith. Proteins and signaling molecules forming an adaptive response to oxidative stress are quite relevant for xenobiotics detoxification and cell protection (Pennington et al., 2005; Landriscina et al., 2009).

One of the most important molecules for superoxide free radical neutralization inside mitochondria is MnSOD. Increased expression of MnSOD correlates with tumor malignancy (Ria et al., 2001; Nozoe et al., 2003; Landriscina et al., 2009; Fu et al., 2016), while moderately elevated expression of MnSOD significantly increases the probability of survival and developing resistance to drugs (Fu et al., 2016).

GSH is of high importance in xenobiotic inactivation and plays an important part in neutralizing anticancer agents, such as cisplatin, carboplatin, and oxaliplatin, in cancer cells (Meijer et al., 1992). GSH carries the active thiol group in the form of a Cys residue and acts as an antioxidant either directly, by interacting with reactive molecules, or as a cofactor of numerous enzymes (Lushchak, 2012). GST protein family catalyzes the conjugation of xenobiotic with GSH, leading to the formation of inactive metabolites that are actively transported by GSH export proteins (including ABCC1 and ABCC2). Moreover, the high expression and activity of GSTπ and other GSH system components are correlated with chemoresistance (Dong et al., 2018; Valente et al., 2021). Increased GSH levels are present in patients who underwent an initial round of treatment with the alkylating drugs cisplatin and doxorubicin (Cui et al., 2018). Platinum-containing drugs are covalently bound to GSH and extruded out of the cell by the ABC pumps. Temozolomide, as an alkylating agent, primarily induces DNA damage and consequently, cell death. An attribute of temozolomide treatment in glioma cells is over-activated antioxidant systems, with high GSH and GR content; furthermore, the suppression of GSH sensitizes glioma cells to temozolomide (Zhu et al., 2018). The silencing of *GRX* expression sensitizes ovarian cancer cells to doxorubicin (Lillig et al., 2004). GSH is also involved in resistance to targeted therapy, such as bortezomib (Starheim et al., 2016) and imatinib (Frank et al., 2006).

Increased activity of the Trx system, as well as its downstream molecules, is involved in the development of resistance to chemotherapy. Overexpression of both Trx and TrxR has been reported in triple-negative breast cancer patients with a poor outcome, while increased Trx expression correlates with aggressive tumors, poor clinical prognosis, and lower patient survival rates in non-small cell lung carcinoma (NSCLC) and colorectal carcinoma (Kakolyris et al., 2001; Raffel et al., 2003; Yoo et al., 2006; Raninga et al., 2020). TrxR2 increased activity is involved in the MDR of wild-type *KRAS* colorectal carcinoma cells (Du et al., 2018). Trx also confers a growth advantage to pancreatic cancer cells and increases their resistance to cisplatin-induced apoptosis (Arnold et al., 2004). A previous study has shown that Trx neutralizes the intracellular toxic oxidants produced by cisplatin leading to the development of drug resistance (Sasada et al., 1996), and that drug-resistant cancer cells are more sensitive to inhibitors of the Trx system (Bjorkhem-Bergman et al., 2002). Increased expression of Trx system proteins also correlates with resistance to doxorubicin, docetaxel, and tamoxifen (Sasada et al., 1996; Kim et al., 2005; Holmgren and Lu, 2010; Karlenius and Tonissen, 2010). In paclitaxel-resistant ovarian cancer cells, Trx1 is involved in overcoming drug toxicity by binding to the FOXO1 transcription factor, inducing its nuclear translocation, and enhancing FOXO1 transcriptional activity (Wang et al., 2015). Trx mediates the resistance to tamoxifen therapy in breast cancer cells through changes in H_2_O_2_ production and modulation of estrogen-dependent and estrogen-independent redox-sensitive signaling pathways (Penney and Roy, 2013). Diffuse large B cell lymphomas derived from primary cell culture, as well as cell lines, express higher basal levels of Trx1 than normal B cells. Moreover, Trx1 expression level is associated with decreased patients’ survival indicating that Trx1 plays a key role in cell growth, survival, and drug resistance (Li et al., 2012).

Previous studies have shown that overexpression of Trx-dependent protein Prx occurs in various human cancers (Fourquet et al., 2008; Kim and Jang, 2019). Namely, Prx1 has proved to be a tumor promoter in numerous types of cancer, by interacting with cancer-associated signal pathways including (I) increased expression of vascular endothelial growth factor (VEGF), (II) activation of c-Jun and AP-1, (III) inhibition of E-cadherin and consequent induction of epithelial-mesenchymal transition, and (IV) suppression of apoptosis through the inhibition of ASK-1 (Nicolussi et al., 2017). Overexpression of Prx2 has also been reported in breast, prostate, and esophageal cancer (Nicolussi et al., 2017). Furthermore, increased expression of Prx proteins is identified as an important cog in the machine of chemoresistance and radioresistance (Chung et al., 2001; Kalinina et al., 2012; Hwang et al., 2013). Elevated Prx1 expression provides resistance to docetaxel treatment in lung cancer cells through the suppression of FOXO1-induced apoptosis (Hwang et al., 2013). Prx2 plays an important role in breast cancer cell resistance to ionizing radiation, while silencing the *PRX2* gene may increase the sensitivity to radiotherapy (Wang et al., 2014). Increased expression of Prx2 inhibits cisplatin-induced apoptosis in a gastric adenocarcinoma cell line, thereby conferring chemoresistance of cancer cells, especially to oxidative stress producing anticancer drugs (Chung et al., 2001). Overexpression of Prx1 and Prx2 contributes to cisplatin resistance in erythroleukemia cell lines as well as in breast and ovarian carcinoma cell lines (Kalinina et al., 2012). Cervical cancer patients with high levels of Trx1, Prx1, and Prx2 have a poor response to cisplatin-based therapy, compared to those with low protein expression (Zhu H. et al., 2019). Moreover, breast cancer cells, resistant to docetaxel, exhibit overexpression of *TRX*, and *PRX* genes (Iwao-Koizumi et al., 2005).

The overexpression of the Nrf2 transcription factor, involved in the regulation of Trx and TrxR expression, leads to the development of drug resistance. Nrf2 expression was significantly higher in cisplatin-resistant and non-responding patients than in good responders (Roh et al., 2017b). Chronic myeloid leukemia (CML) cancer cells, resistant to imatinib, express higher levels of Nrf2 and TrxR, involved in protecting cancer cells from the harmful effects of the drug (Xu et al., 2019). Silencing of Nrf2 sensitizes the CML imatinib-resistant cells, ovarian cisplatin-resistant cancer cells, and lung doxorubicin-resistant cancer cells (Cho et al., 2008; Xu et al., 2019). Mutations in the *KEAP1* gene, causing destabilized Nrf2/Keap1 relation and excessive Nrf2 activity, are associated with occurrence and chemoresistance in leukemia, lung, breast, colon, gastric, and liver cancer (Padmanabhan et al., 2006; Hayes and McMahon, 2009; Rushworth and Macewan, 2011; Yoo et al., 2012; Ganan-Gomez et al., 2013).

## Trx System Inhibition in Cancer Treatment and Overcoming Drug Resistance

Inhibition of TrxR reduces cancer cell survival upon the attack of anticancer agents and sensitizes cancer cells to chemotherapeutics (Lee et al., 2019; Jovanovic et al., 2020a). Moreover, inhibition of TrxR results in an increased concentration of oxidized Trx and affects the whole Trx system, ultimately increasing RONS concentration and causing apoptosis, necrosis, and autophagy in different model systems (Galadari et al., 2017; Jovanovic et al., 2019; Lee et al., 2019). Though Trx has been considered in past as a potential target for drug development, TrxR is more often the focus of the development of Trx system inhibitors (Karlenius and Tonissen, 2010). A heap of evidence shows that numerous cytostatics, including carmustine, cisplatin, motexafin gadolinium, and arsenic trioxide, have inhibitory potential toward TrxR, and that the anticancer effect of these drugs can be at least partly ascribed to TrxR inhibition (Cai et al., 2012). Some of the mentioned drugs have multiple targets in the antioxidant system; for example, besides TrxR, arsenic trioxide targets GPx and SOD as well (Li et al., 2006). Several natural compounds, with tumor-suppressive effects, such as curcumin, piperlongumine, green, and black tea extracts, are also inhibitors of TrxR function (Karlenius and Tonissen, 2010; Cai et al., 2012; He et al., 2019; Zhang et al., 2019). Oleanolic acid is a natural product, widely distributed in plants. OLO-2, an oleanolic acid derivative, targeting both TrxR activity and expression, synergizes with cisplatin in lung cancer-cisplatin-resistant cells and increases apoptotic response (Zhu B. et al., 2019). By decreasing the activity of PI3K/Akt and NF-kB signaling pathways, OLO-2 consequently decreases the expression of ABCB1 in resistant cells as well. Small electrophilic molecules, such as recently reported Ugi-type Michael acceptors (UMAs), are efficient inhibitors of TrxR1, with an anti-tumor effect (Jovanovic et al., 2019; Jovanovic et al., 2020b). UMAs, specific TrxR1 inhibitors, are selective toward cancer over normal cells, induce cell death by elevating RONS, suppress the invasive and migratory potential of glioma cells, and sensitize glioma cells to temozolomide (Jovanovic et al., 2019; Jovanovic et al., 2020a; Jovanovic et al., 2020b).

Auranofin, an anti-rheumatic in clinical use (Roder and Thomson, 2015), having TrxR as a primary molecular target, is indicated as a candidate for the supplementation of chemo- and radiation therapy (Sachweh et al., 2015; Li et al., 2016; Rodman et al., 2016; Hou et al., 2018; Parrales et al., 2018; Fidyt et al., 2019; Cirri et al., 2020). (Raninga et al., 2020) demonstrated that auranofin inhibits the growth of triple-negative breast cancer cells, *in vitro* and *in vivo*–on account of boosting up the expression of PD-L1. Further on, it has been shown that the anti-inflammatory drug celecoxib synergizes with auranofin and enhances its therapeutic effect in colorectal cancer cells showing a selective cytotoxic effect towards cancer cell lines when compared with normal cells (Han et al., 2019). Platinum-based anticancer drugs (e.g., oxaliplatin and cisplatin), like alkylating agents causing DNA damage, are unselective toward cancer cells, and cause side effects, impairing significantly patients’ quality of life. Therefore, it has been suggested that auranofin, as a gold-based compound, might be a more suitable anticancer drug than platinum-based compounds (Marmol et al., 2019). In imatinib-resistant gastrointestinal stromal tumor cells, auranofin causes significant oxidative stress, leading to a substantial reduction in cell growth and viability (Pessetto et al., 2013). Simultaneous inhibition of TrxR1 by auranofin, and Akt signaling pathway by MK2206, induced significant ROS accumulation, JNK activation, and PARP cleavage, leading to an Nrf2/Keap1-dependant lung cancer cell apoptosis (Dai et al., 2013). What’s more, the anticancer effect of auranofin might be enhanced by the simultaneous treatment with Sec, a crucial amino-acid of the TrxR catalytically active domain (Fan et al., 2014). Auranofin, as an FDA-approved drug, might be of significant value in the clinic, in particular for patients with recurrent disease, with MDR-triggered cancer cells.

Members of the organoselenium class of compounds are also effective inhibitors of TrxR. Among them is ethaselen (BBSKE), a well-described and much studied TrxR inhibitor, with an anticancer effect in different types of cancer cells (Zhao et al., 2006; Xing et al., 2008; Wang et al., 2012). A study by (Wu et al., 2020) demonstrated that ethaselen, as an inhibitor of TrxR, is consequently efficient in reducing the growth of patient-derived, gastric cancer cells organoids, inducing apoptosis of cancer cells as well. The authors reported concentration-dependent induced apoptosis, along with decreased expression of caspase 3, indicating a caspase-independent cell death mechanism of action. Butaselen, another member of the organoselenium class of compounds, impairs the progression of hepatocellular cancer in mice models, causing inhibition in activity of TrxR, as well as causing lower expression of the enzyme, ultimately increasing ROS (Zheng et al., 2018). As the authors highlighted, inhibition of TrxR and Trx by butaselen consequently inhibited the NF-kB pathway, an oncogenic signaling mechanism previously activated by carcinogenesis in transformed cells.

Another approach to inhibiting Trx system activity is by increasing TXNIP expression. d-allose, by itself a non-toxic aldohexose, inhibits the proliferation of head and neck cancer cells by inducing the expression of TXNIP (Hoshikawa et al., 2010). In combination with radiotherapy, increased TXNIP expression induces the generation of cellular RONS and triggers cell death (Hoshikawa et al., 2011), while d-allose synergizes the docetaxel cytotoxic effect, by increasing TXNIP and RONS (Indo et al., 2014). Both inhibition of Trx by PX-12 and overexpression of TXNIP restored sensitivity to cisplatin and sensitized glioma cells to temozolomide (Haas et al., 2018).

However, in some cases and cancer types, Trx system inhibitors showed discouraging results, with little to no effect on tumor growth and progression (Ramanathan et al., 2011). As it turned out, the lack of response to the inhibitor was mainly due to the low target expression. Therefore, the determining factor in the selectivity of Trx/TrxR inhibitor toward cancer cells is the redox phenotype of the cell, which roughly can be identified as the level of expression in redox system proteins. Further on, as mentioned earlier, the GSH system is an important part of cellular redox balance as well. Inhibition of the Trx system can lead to an increase in GSH system activity and prevent the anti-tumor effect. Thus, in defeating cancer through redox systems tempering, dual inhibition of both GSH and Trx systems proved to be more effective than inhibition of individual systems (Sobhakumari et al., 2012; Benhar et al., 2016; Rodman et al., 2016; Roh et al., 2017a). Recent research provided evidence that there is another mechanism of resistance to Trx system inhibitors, mediated by hydrogen sulfide (H_2_S) (Mao et al., 2019; Mao et al., 2020). H_2_S is endogenously produced from l-cysteine, with versatile biological functions–this gaseous mediator has a direct antioxidant effect, by scavenging free radicals, and by promoting other proteins of antioxidant machinery as well, such as GSH, SOD, catalase, and Trx. Tumor cells with high activity of H_2_S-synthesizing enzyme cystathionine γ-lyase are more resistant to the cytotoxic effect of auranofin (Mao et al., 2019) and PX-12 (Mao et al., 2020). H_2_S promotes a reduced state of Trx, and directly interacts with and inactivates some inhibitors. Additionally, H_2_S induces sulfhydryl residues in proteins to compete with Trx for PX-12 binding, thus reducing the available PX-12 for Trx inhibition (Mao et al., 2020). Following the above mentioned, abundance of H_2_S in cancer cells could be an important prognostic factor of Trx system inhibitors’ efficacy.

## Clinical Trials With Trx and TrxR Inhibitors

Trx system is composed of important molecular targets for drug development and a significant amount of evidence in preclinical research supports the statement. Notwithstanding, only a few inhibitors of the Trx system (PX-12, as an inhibitor of Trx, auranofin, and ethaselen as inhibitors of TrxR) have entered clinical trials, so far.

Phase I of the clinical study with PX-12, with 38 advanced, solid tumor patients, refractory to standard therapies, showed that the drug was tolerated up to 226 mg/m^2^ when given by a 3 h intravenous (IV) infusion on days 1–5, repeated every 3 weeks (Ramanathan et al., 2007). PX-12 administration resulted in disease stabilization in seven patients (126–332 days) when lower than the maximally tolerated doses were applied (Ramanathan et al., 2007). The limiting factor of dose increase was the strong odor, caused by the evaporation of PX-12 metabolite–2-butanethiol. Later on, another Phase I of prolonged IV infusion PX-12 schedule has been administered, in 14 patients with advanced or metastatic cancer (NCT00736372, 2008). PX-12 at a dose of 400 mg/m^2^/day by a 72-h infusion, over days 1, 2, and 3 in a 21-days cycle was safe and tolerable (Ramanathan et al., 2012). Trial phase Ib included patients with advanced gastrointestinal cancers, with an IV 24-h protocol; the maximally tolerated dose reported was 300 mg/m^2^/24 h, once per week. At this dose, the 2-butanethiol odor was bearable (Baker et al., 2013). The pharmacokinetics of PX-12 demonstrated rapid, irreversible binding to plasma components, resulting in low plasma concentrations of non-bound PX-12 during infusion (Baker et al., 2013), while the concentration of the inactive metabolite, 2-mercaptoimidazole, increased linearly with PX-12 dose escalation (Ramanathan et al., 2007).

Phase II with PX-12 was designed as a randomized study with 17 patients, with advanced pancreas cancer, showing signs of disease progression after a gemcitabine-containing combination of drugs. The participants in the study received PX-12 (54 or 128 mg/m^2^) by a 3-h IV, for 5 days in a 21-days cycle (NCT00417287, 2007). PX-12 was well tolerated, and severe adverse events were rare. The best response was stable disease in two patients, while none of the patients had a progression-free survival over 4 months (Ramanathan et al., 2011).

The clinical trials evidenced that PX-12 causes target inhibition, due to the decrease of Trx-1 level in plasma, but only if Trx-1 plasma level in patients, before treatment, was above a certain threshold (for gastrointestinal cancer >18 ng/ml) (Ramanathan et al., 2012; Baker et al., 2013). From the performed clinical trials, it was concluded that PX-12 adds to disease stabilization in some patients (Ramanathan et al., 2007; Ramanathan et al., 2011; Ramanathan et al., 2012). Succeeding trial phases for PX-12 were terminated, due to the demanding administration (continuous IV infusion) and anticipated severe side effects in a long-run application, particularly pneumonitis. However, the Trx-1 pathway remains a target of interest in patients with advanced and refractory malignancies. The development of the next generation of inhibitors is much needed, implemented in studies with improved patient stratification (i.e., according to Trx-1 plasma level).

Auranofin, an FDA-approved drug for treating rheumatoid arthritis since 1985 (Roder and Thomson, 2015), has recently been investigated in Phase I/II clinical trial, in combination with sirolimus (rapamycin, an mTOR inhibitor). The study was conducted with NSCLC (squamous or *RAS*-mutated adenocarcinoma) and small cell lung carcinoma patients, with metastatic or relapsed disease, that cannot be controlled with standard treatment regimens (NCT01737502, 2012). The status of this study is “still recruiting” and 47 patients were estimated to enter the study, over 10 years, since 2012. The clinical trial is sponsored by Mayo Clinic, in collaboration with National Cancer Institute (NCI), United States. The study completion is expected in August 2022. The primary goal in Phase I is to establish the maximum-tolerated dose of auranofin in combination with sirolimus, after at least one line of platinum-based chemotherapy, while the primary goal of Phase II is to achieve the progression-free survival of 4 months in patients treated with auranofin, after at least one line of platinum-based chemotherapy.

Auranofin was recently included in a clinical trial Phase I, with 10 recurrent glioblastoma patients (NCT02770378, 2016). In fact, the mentioned clinical trial was a proof-of-concept, assessing the safety of the coordinated undermining of survival paths by 9 repurposed drugs (CUSP9v3), combined with temozolomide metronomic treatment (Halatsch et al., 2021). The clinical study, sponsored by the University of Ulm, in collaboration with Reliable Cancer Therapies and Anticancer Fund, Belgium, was completed in December 2020. Treatment consisted of the following repurposed drugs: 1) aprepitant, an inhibitor of neurokinin-1 (Akazawa et al., 2009), 2) auranofin, as an inhibitor of TrxR, 3) celecoxib, a cyclooxygenase-2 inhibitor (Stockhammer et al., 2010), 4) captopril, an MMP-2 and MMP-9 inhibitor (Rooprai et al., 2001), 5) disulfiram, an aldehyde dehydrogenase inhibitor (Wang and Darling, 2013), 6) itraconazole, an antifungal agent which inducer of autophagy through abnormal cholesterol trafficking (Liu et al., 2014), 7) minocycline, an antibiotic agent that inhibits MMP-9 and Toll-like receptor 2 (Hu et al., 2014), 8) ritonavir, an antiretroviral drug that induces endoplasmic reticulum stress (Rauschenbach et al., 2020) and 9) sertraline, an antidepressant which inhibits ABCB1 in blood-brain barrier (O'Brien et al., 2012), and continuous, metronomic administration of low-dose temozolomide. The treatment started with temozolomide (20 mg/m^2^) and aprepitant (80 mg per day) on day 1, followed by the addition of one drug every 2 days (day 3, day 5, etc.) at the low-dose level. Auranofin was the last drug in line, added on day 17. On day 19, the up-dosing phase started with only one drug dose being increased every 2 days. During the study, ritonavir, temozolomide, captopril, and itraconazole required dose modification or pausing. According to the results obtained on nine evaluable glioblastoma patients, there was a 50% 12-months progression-free survival. Application of auranofin in combination with other drugs led to ALAT (liver enzyme) increase in four patients, lymphocyte count decreased in eight patients, white blood cell decrease in one patient, and nausea in one patient. Phase I clinical trial showed that CUSP9v3 can be safely administered under careful monitoring. A randomized Phase II clinical trial will assess the efficacy of the CUSP9v3 regimen in glioblastoma (Halatsch et al., 2021).

Clinical trials conducted in China have primarily been focused on ethaselen. Phases 1a and 1b showed that 1200-mg ethaselen per day is a well-tolerated dose in NSCLC patients, harboring a high TrxR expression, confirmed by immunohistochemistry (Wang et al., 2011; Wang et al., 2012). The following Phase 1c was recently completed (December 2020) but the results have not been published yet. The study included 40 patients with advanced NSCLC patients having received more than two lines of standard therapy. The study was financed by Hunan Province Tumor Hospital, China, and it lasted for 6 years (NCT02166242, 2014). Patients received the oral, dispersible tablet of ethaselen, 600 mg twice per day. The primary endpoint of the study was a 6-weeks disease control rate according to complete response, partial response, and stable disease, while secondary endpoints included progression-free survival, overall survival, quality of life, and drug safety.

Clinical studies encompassing Trx system inhibitors have been rare in the past. Yet, clinical development of TrxR inhibitors might be taking a new turn, with several promising studies being launched recently. Auranofin is perceived as a more selective metal-containing anticancer agent, with fewer side effects, compared to platinum-based drugs (Marmol et al., 2019). Both auranofin and ethaselen were included in clinical trials with NSCLC patients, who previously received at least one line of chemotherapy with platinum-based drugs (NCT01737502, 2012; NCT02166242, 2014). Particular progress is envisioned regarding combined treatment options with auranofin in the therapy of advanced malignancies, such as NSCLC and glioblastoma (NCT01737502, 2012; NCT02770378, 2016).

## Conclusion

Redox balance disturbance has been identified as the Achilles’ heel of cancer cells, and this weakness should be taken into consideration in cancer treatment evolution (Jia et al., 2019). More than half a century of research on Trx and TrxR in the field of cancer biology fortified the hypothesis that the Trx system is of utmost significance in cancer cells’ survival, proliferation, invasion, and metastasis (Gromer et al., 2004; Arner and Holmgren, 2006; Holmgren and Lu, 2010; Jia et al., 2019). Apart from preserving the cells and promoting cancer growth, the Trx system plays a strong role in the detoxification of xenobiotic drugs as well. In this review, we discussed some studies where the highly active Trx system proved to contribute to drug treatment-poor response. The attributes of this redox regulating system make it an attractive target for chemotherapy drug development. The core strategy of Trx system inhibitors usage is an increase in oxidative stress, cancer cell damage beyond repair, and cell death induction. Some cytostatics approved for cancer treatment inhibit the Trx system non-specifically, and the therapeutic effect is ascribed in part to Trx system impairment. Despite intensive development of new and specific compounds–inhibitors of Trx or TrxR and promising results in preclinical studies, very few inhibitors have come to clinical trials. None of these inhibitors had been approved for the clinic so far. In developing novel anticancer therapies employing Trx system inhibitors, future research should focus on overcoming some of the key obstacles of the treatment, such as compensation of redox regulation by the GSH system. In Trx system inhibitors research for cancer treatment, more attention should be given to the bioavailability of the drugs, and before suggesting prospective candidates. Further, the exploration of indirect, cancer-specific inhibition of the system should be expanded as a possibility. Manipulation of antioxidant defense proved to have case-to-case variable results, with a tumor-suppressive outcome, synergizing with standard clinical therapy approaches in some types of tumors and stages, to having no effect or even aggravating tumor progression and metastasis (Acharya et al., 2010; Sotgia et al., 2011; Sayin et al., 2014; Piskounova et al., 2015; Buric et al., 2019). Of crucial importance for clinical trials with Trx system inhibitors must be clear evidence of Trx system proteins over-expression and/or elevated activity in classified cancer patients to have rational bases for inhibitor testing.

## Glossary

ABC: ATP-binding cassette
ABCB1: ATP-binding cassette subfamily B member 1
ABCC1 and ABCC2: ATP-binding cassette subfamily C member 1 and 2
ABCG2: ATP-binding cassette subfamily G member 2
AML: acute myeloid leukemia
ARE: antioxidant response element
ASK1: apoptosis signal-regulating kinase 1
AP-1: activator protein 1
CAT: catalase
CML: chronic myeloid leukemia
Cys: cysteine
DNA: deoxyribonucleic acid
GBM: glioblastoma
GPx: glutathione peroxidase
Grx: glutaredoxin
GR: glutathione reductase
GSH: reduced glutathione
GSSG: oxidized glutathione
GST: glutathione-S-transferase
JNK: c-Jun N-terminal kinase
Keap1: Kelch Like ECH Associated Protein 1
MAP: microtubule-associated protein
NADPH: nicotinamide adenine dinucleotide phosphate
Nrf2: nuclear factor erythroid 2-related factor 2
NSCLC: non-small cell lung carcinoma
Prx: peroxiredoxin
RNR: ribonucleotide reductase
RNS: reactive nitrogen species
RONS: reactive oxygen and nitrogen species
ROS: reactive oxygen species
Sec: selenocystein
SOD: superoxide dismutase
Trx: thioredoxin
TrxR: thioredoxin reductase
TXNIP: thioredoxin-interacting protein
UMA: Ugi-type Michael acceptor
